# THE IMPACT OF ENVIRONMENTAL EXPOSURES ON DEMENTIA, PHYSICAL, COGNITIVE, AND MENTAL HEALTH OF OLDER ADULTS

**DOI:** 10.1093/geroni/igae098.1248

**Published:** 2024-12-31

**Authors:** Paola Zaninotto, Matthew Prina

**Affiliations:** Chair; Discussant

## Abstract

The proposed symposium will delve into crucial environmental factors affecting physical health, cognitive and mental health, in older populations. The first talk will explore urban environments’ impact on cognitive health, highlighting evidence gaps and concentrated areas of existing research, such as air pollution and social environment influences on dementia and Alzheimer’s disease. Another presentation will examine the association between long-term air pollution exposure and dementia risk in Denmark, identifying the most vulnerable groups based on socioeconomic status and comorbidities. The third talk will focus on the relationship between air pollution exposure over a decade and cognitive performance in older individuals in England, emphasizing the detrimental effects of industry emissions on cognition. A different study will discuss the findings on outdoor light at night and its lack of association with depressive symptoms in older adults in England. Lastly, the symposium will present research on extreme heat exposure’s effects on epigenetic aging and its potential to increase morbidity and mortality risks, underscoring the need for mitigating these biological changes.

